# Gastrointestinal acute radiation syndrome: current knowledge and perspectives

**DOI:** 10.1038/s41420-025-02525-6

**Published:** 2025-05-14

**Authors:** Michael L. Freeman

**Affiliations:** Department of Radiation Oncology, Vanderbilt University Medical Center, Nashville, TN USA

**Keywords:** Pluripotent stem cells, Trauma

## Abstract

Acute radiation gastrointestinal syndrome (GI-ARS) develops when the intestine is rapidly exposed to large doses of ionizing radiation. In humans, GI-ARS occurs at radiation doses of 6 Gy, with doses of ≥10 Gy typically resulting in death within 10 days. This condition can be caused by various factors, including war, terrorism, nuclear power plant accidents, and cancer therapy-associated adverse events. Developing effective approaches for treating GI-ARS requires a comprehensive understanding of the syndrome. This review summarizes the current body of literature that defines GI-ARS as a consequence of intestinal irradiation. It highlights the paradigm shift in understanding which intestinal stem cells contribute to homeostasis, the critical role of vascular injury in the development of GI-ARS, and recent advances in research on crypt-villus regeneration following radiation injury.

## Facts

- Radiation-induced Gastrointestinal Syndrome develops in the intestine following acute exposure to ionizing radiation doses of ≥6 Gy (≥10 Gy is fatal).
- Radiation-induced damage to endothelial cells within the lamina propria contributes to the development of this syndrome.
- Recent studies have revealed a paradigm shift in the location of intestinal stem cells. These cells, referred to as isthmus progenitor cells, are now recognized as critical contributors to crypt-villus homeostasis and regeneration.
- Mitotic catastrophe is the primary cell death mechanism leading to the loss of viability of crypt cells responsible for the crypt-villus regeneration.

## Questions

- What are the molecular mechanisms responsible for vascular regeneration in the lamina propria following irradiation?
- What signals/cytokines/chemokines are generated by the irradiated cells that stimulate isthmus progenitor cells to regenerate crypts?
- How do age and sex influence the mechanisms driving regeneration after radiation exposure?
- Are Lgr5+ crypt base columnar cells more radiosensitive than isthmus progenitor cells?

## Introduction

Acute radiation syndrome (ARS) is a condition that develops when an individual is exposed to very high doses of ionizing radiation. Several criteria must be met for the onset of ARS: a considerable portion of the body must absorb the radiation dose, the radiation must penetrate internal organs, and the dose must be delivered over a very short period (minutes or hours). The U.S. Centers for Disease Control and Prevention (CDC) identifies three classic syndromes associated with ARS: hematopoietic, gastrointestinal, and neurovascular (https://www.cdc.gov/radiation-emergencies/hcp/clinical-guidance/ars.html). The initial stage of each syndrome is termed the prodromal stage, during which clinical symptoms develop. The larger the dose, the more rapidly this stage begins. This is followed by the latent stage, which is the interval during which symptoms subside. The next stage is the illness manifestation stage, in which symptoms reappear, varying in severity depending on the syndrome and radiation dose. Death or recovery occurred during the final stage. The neurovascular syndrome, otherwise known as cerebrovascular syndrome, develops rapidly following whole-body exposure to 100 Gray (Gy) or more. Death typically occurs within 24–48 h. Gastrointestinal (GI) syndrome develops at radiation doses of 6–12 Gy. At doses of ≥10 Gy, death generally occurs within 9–10 days. Doses as low as 2.5 Gy induce the hematopoietic syndrome, with death occurring within 30–60 days [1]. The mean lethal radiation dose (LD_50_) for humans is estimated to be 3.5–4 Gy without supportive care and 4.5–7 Gy with intensive medical intervention [2]. Several comprehensive reviews of ARS have been conducted [1, 3]. This study focuses on GI syndrome, one of the most severe forms of ARS.

The earliest documented report of GI injury caused by ionizing radiation that we are aware of dates back to 1897 in a publication by David Walsh, M.D [4]. Dr. Walsh described a case in which a male researcher underwent daily 2-h X-irradiation of the “stomach” region and subsequently experienced “gastric symptoms, such as pain, tenderness, flatulence, colic, and diarrhea.” He observed that placing a lead sheet over the abdomen prevented the onset of symptoms. Dr. Walsh interpreted these events as evidence that X-irradiation “caused a direct inflammation of the gastro-intestinal mucous membranes.” However, the question of whether X-irradiation could actually cause organ injury was not widely accepted in the early years of its use for diagnostic or therapeutic purposes [5], and little attention was given to tissue injury. In 1906, David Edsall, M.D., critiqued the unregulated application of X-rays, stating that practitioners often applied the technology “for both diagnostic and therapeutic purposes in all manner of conditions, only to gradually, largely through painful experience, to learn that it may have dangerous adverse effects” [5]. As the use of X-irradiation for therapeutic purposes increased, so did a condition known as “treatment sickness” [6]. Animal studies were performed aimed at characterizing tissue sensitivity and symptom severity. Denis et al. [6] and Regaud et al. [7] used animal models of irradiation injury to demonstrate that ionizing radiation can cause intestinal injury. However, it was not until the experiments undertaken by Warren and Whipple [8] that the primary mechanism of injury resulting from abdominal irradiation was correctly identified as the loss of the intestinal epithelium, which “covers the villi and lines the crypts.”

The understanding of acute radiation effects expanded significantly during World War II following the atomic bombings of the Hiroshima and Nagasaki cities in Japan by the United States on August 6th and 9th, 1945. Soon after, Colonel P.D. Keller, M.D., of the U.S. Army Medical Corps, examined 11 Japanese patients at Osaka University Hospital and reviewed hospital records for an additional 10 patients diagnosed with what was termed atomic bomb disease [9]. His landmark report was developed from the data of these patients [9], including 18 men and three women, with an average age of 30 years (20–52 years). Patients exposed to the atomic blasts experienced nausea, vomiting, and anorexia. The onset and duration of these symptoms vary based on their proximity to the epicenter of the explosion. Some individuals developed symptoms within 30 min of the explosion, whereas others experienced delays of 1–2 days. Additional symptoms included fatigue, diarrhea, and leukopenia. The concept that acute total body irradiation could result in a specific syndrome, now recognized as ARS, was elucidated by Lieutenant Commander E.P. Cronkite, M.D., and Lieutenant W.H. Chapman, M.D., USN [10]. Based on their observations of atomic bomb survivors in Japan, they described an initial syndrome characterized by nausea, vomiting, diarrhea, and malaise developing within hours of exposure to radiation following the absorption of 600–1500 roentgen [10]. The symptoms increased with increasing radiation dose. The latent period followed the initial syndrome, and its duration was inversely proportional to the radiation dose. The latent period was defined “as the interval between the subsidence of the initial symptoms and the recurrence of symptoms, predominantly manifested as GI problems, such as intractable and bloody diarrhea, purpura, fever and pancytopenia” [10]. At the highest dose, death occurred within 4–10 days. Based on this study and subsequent research [11–13], ARS has been formally categorized into four stages: prodromal, latent, manifestation, and death or recovery [1].

## The pathophysiology of GI-ARS

**Fig. 1:**
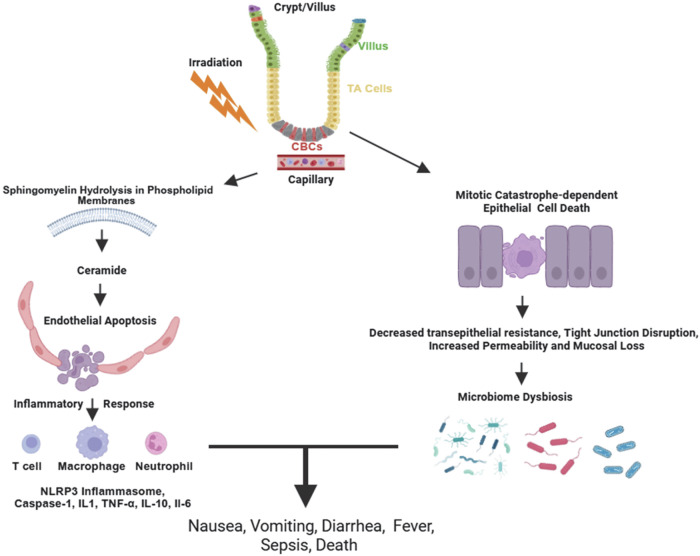
An overview of gastrointestinal acute radiation syndrome (gi-ars) pathophysiology. Following absorption of ionizing radiation, membrane sphingomyelin undergoes hydrolysis, resulting in the generation of ceramide that drives endothelial cell apoptosis. An inflammatory response follows, consisting of inflammatory cells and cytokines, such as IL-1, TNF-α, IL-10 and IL -6. Ionizing radiation also results in epithelial cell death due to mitotic catastrophe. There is loss of transepithelial resistance and disruption of tight junctions leading to microbiome dysbiosis. Nausea, vomiting, diarrhea, fever sepsis and death can follow. Developed using BioRender.

Currently, there are no Food and Drug Administration (FDA) approved countermeasures for GI-ARS in the event of radiological/nuclear terrorism, radiological accidents, or radiation therapy-induced GI injury. To develop effective countermeasures, it is essential to conduct extensive research aimed at achieving a comprehensive understanding of the pathophysiology of radiation-induced intestinal injury and the subsequent processes of regeneration [14]. Within just 10 ms of a cell absorbing ionizing radiation (photons or high linear energy particles such as neutrons), DNA and other biomolecules are damaged [1]. Depending on the dose absorbed, this damage triggers a cascade of biological responses, including sphingomyelin hydrolysis and ceramide generation [15–17], epithelium apoptosis, endothelial apoptosis and mitotic catastrophe, infiltration of inflammatory cells into intestinal crypts [18], activation of Caspase 1, increased expression of pro-inflammatory cytokines such as interleukin IL1β, and tissue necrosis factor TNF-α, and the expression of NOD-like receptor protein 3 (NLRP3) [19–21]. These events collectively contribute to a decrease in transepithelial resistance due to the loss of the epithelial tight junction barrier (Fig. 1) [22]. While crypt regeneration may occur, the likelihood of successful regeneration decreases as the radiation dose increases [23]. It is estimated that a dose as low as 6 Gy to the human abdomen can induce GI-ARS symptoms [24], while doses exceeding 10 Gy result in fatal GI-ARS. Two outstanding and comprehensive reviews of GI-ARS countermeasures have been published [25, 26], and rather than reiterating the information contained within those papers, readers are encouraged to consult these reviews directly for a more comprehensive understanding of the topic.

## Crypts of Lieberkuhn—structure and renewal

**Fig. 2:**
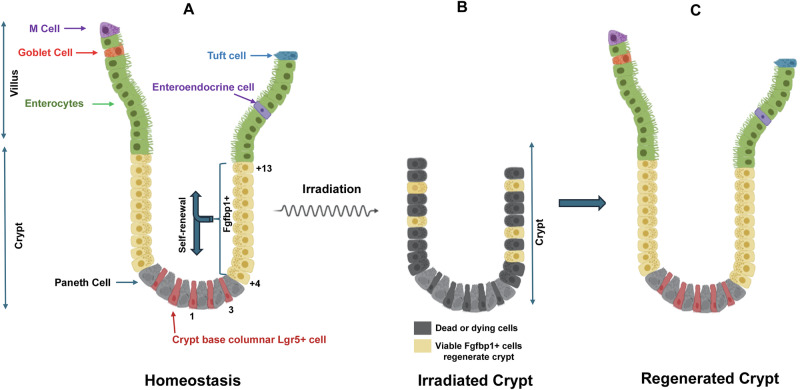
Illustration of crypt-villus structures. **A** Multipotent Fgfbp1-mediated homeostatic self-renewal, generating both CBC Lgr5+ and multi-lineage intestinal cells; **B** Example of an irradiated crypt with surviving multipotent Fgfbp1 cells capable of crypt regeneration. Cells colored black are dead or dying cells; **C** A crypt-villus regenerated from surviving multipotent Fgfbp1 cells. Developed using BioRender.

The small intestine is intricately organized into structures known as plicae circulares, or Kerckring folds, and is divided into the duodenum, jejunum, and ileum [27, 28]. Within these regions are various cell types, including Paneth, crypt-based columnar (CBC), transit-amplifying, enterocytes, goblet, enteroendocrine, tuft and M cells (Fig. 2A) [29]. Paneth cells are located at the bottom of crypts, have a pyramid-like shape and secrete antimicrobials. CBCs are also located in the bottom of crypts, in between Paneth cells. They are Lgr5+ self-renewing cells that generate transit-amplifying (TA) cells. TA cells are progenitor cells located along crypt positions +4 to +13 (Fig. 2A) that differentiate into various lineages. Enterocytes are differentiated brush-border, polarized columnar cells that are attached to each other via tight junctions, with a carbohydrate glycocalyx on the surface. A major role is nutrient absorption. Goblet cells are also differentiated columnar cells that secrete mucins [30]. Differentiated enteroendocrine cells secrete peptide hormones in response to microbes [31]. Tuff cells are chemosensory cells designed to detect microorganisms and then inform effector cells [32]. Microfold (M) cells are phagocytotic, taking up luminal bacteria and antigens, and informing dendritic cells [33]. Lamina propria is a connective tissue containing blood and lymphatic vessels and immune cells that surrounds crypts [34]. The regeneration of crypt-villus occurs every 3–5 days because of the high rate of crypt cell proliferation, which is balanced by the shedding of fully differentiated villi [35–37]. In mice, there are approximately 200 cells per crypt, with about one million intestinal crypts [38]. The numbers are similar in the human.

Johannes N. Lieberkuhn is credited with the seminal description of the intestinal structures in his work Dissertatio Anatomico Physiologica de Fabrica et Actione Villorum Intestinorum Tenuium Hominis [39]. He provided remarkable detailed anatomical observations of the villi that extend from the apical surface of the small intestine and described the invaginations at the base of the villi. These invaginations, known as crypts, function as glands secreting digestive juices [40]. Lieberkuhn’s research also described the arterioles and capillary plexus that support the crypts, now referred to as the lamina propria [40].

Given the rapid rate of self-renewal of the small intestine, studies have focused on identifying the mechanisms underlying crypt regeneration under homeostatic conditions. Leblond and Stevens [41] addressed the question of crypt regeneration by quantifying mitosis in rat intestines, demonstrating that 99.63% of the 1606 mitotic cells observed were localized within crypts. This study was extended using radioisotopes to label rapidly dividing cells. The use of ^32^P, ^35^S-methionine, and ^3^H-thymidine demonstrated that the cells initially labeled were located in the crypts, whereas the radiolabeling of villus cells required longer post-labeling intervals. They concluded that crypts contained putative stem cells that differentiated and migrated upward through the villi, although their exact anatomical locations remained unconfirmed [42–44]. In further studies using ^3^H-thymidine infusions in mice, Cheng and Leblond [45] concluded that all cells in the crypt originated from crypt-based columnar cells, which they identified as stem cells. Crypt-based columnar cells, located at the bottom of the crypts, were immature and labeled with ^3^H-thymidine. Based on the migration of these labeled cells toward the villus tips, Cheng and Leblond hypothesized that crypt-based columnar cells played a crucial role in crypt-villus regeneration as they were pluripotent and underwent terminal differentiation into cells of all lineages [45]. Strong support for this hypothesis emerged from investigations using a chemical mutagenesis approach [46, 47]. These findings established that daughter cells emanating from the crypt-based columnar cells migrated upwards through the crypt and mid-crypt region, commencing differentiation before moving to the villus, where the differentiation was completed [45–47]. However, the exact nature of the crypt stem cells, their precise locations, and the genes expressed by these cells remained unresolved. Radioisotope labeling experiments that quantified cell migration velocity as a function of crypt position provided strong, albeit indirect, evidence that, under homeostatic conditions, crypt stem cells were located at the +4 cell position [36]. Advances in knowledge were made when Korinek et al. [48], discovered that the expression of Tcf-4, a transcription factor that forms a complex with β-catenin in the Wnt signaling pathway, was crucial for maintaining crypt stem cells. Clevers and colleagues [49] posited that the Wnt target gene Lgr5 [50], expressed in cells at the base of the crypt but not in villus cells, could serve as a marker for crypt stem cells. To test this hypothesis, Barker et al. generated Lgr5-LacZ, Lgr5-EGFP-IRES-creERT2, and Rosa26-stop-lacZ knock-in mice [49]. They discovered that the expression of LacZ and EGFP occurred at the base of the crypts in wedge-shaped cells between the Paneth cells, incorporated 5-bromodeoxyuridine and exhibited Ki67 immunopositivity. The use of LacZ expression for lineage tracing demonstrated that Lgr5+ cells were located at the base of a crypt, between Paneth cells, and migrated up through the crypts into villi to generate all terminally differentiated epithelial lineages [49]. Further experiments identified Ascl2 and Olfm4 as Lgr5+ stem cell markers [51]. These observations reinforced the concept that stem cells located at the base of the crypt allow for “continuous linear migration of cells from the lower crypt upward to the luminal surface” [36] and highlighted the importance of Wnt-mediated signaling in this process. Recently, the Frizzled receptor FZD5 has been identified as essential for Lgr5-dependent crypt cell homeostasis in the duodenum, jejunum, and ileum [52, 53].

Currently, six hallmark characteristics of stem cells have been described [54]: self-renewal, multipotency, plasticity, maintenance of genome integrity, transplantability, and niche dependence. A comparison of these hallmarks with Lgr5+ cell biology aligns with the concept that Lgr5+ serves as a primary stem cell marker of intestinal homeostasis. However, recent studies have elucidated a more nuanced understanding of the intestinal stem cells paradigm: Lgr5^low^ cells located along the isthmus (+4 through +13) exhibit the highest degree of stemness, multi-potency and the ability to give rise to Lgr5+ cells during homeostasis and following injury (Fig. 2A–C) [37, 55]. Malagola et al. [55]. applied an unbiased single-cell RNA sequencing\VIPER approach combined with analyses of crypt epithelial cell chromatin accessibility via a single-cell sequencing assay for transposase-accessible chromatin (scATAC-seq) to identify cells characterized by inferred stemness potency; Fgfbp1 and Lgr4 were identified as genes associated with high stem cell potency, not Lgr5, and were found in cells located in positions +4 to +13. These were termed “isthmus progenitor” cells and were shown to expand bi-directionally and function as both homeostatic and rescue stem cells. Strikingly, Lgf5 expression was not associated with the highest stemness potential; rather, it was associated with differentiated cells, as well as with crypt base columnar (CBCs). Capdevila et al. also identified Fgfbp1-expressing isthmus cells using a kinetic reporter for time-resolved fate mapping and Fgfbp1-CreER^T2^ lineage tracing [37]. They too demonstrated that isthmus cells function as homeostatic and rescue stem cells that exhibit bidirectional propagation. Importantly, “isthmus progenitor” cells were able to grow as organoids [37, 55]. Although Lgr5+ cells may not exhibit the highest degree of stemness, Lgr5 expression is indispensable for crypt regeneration following irradiation [56]. Further research is essential to provide a comprehensive understanding of small intestinal biology.

## Role of ceramide in GI-ARS

Ceramides are bioactive lipids synthesized via two major pathways. One is a de novo pathway, and the second involves the catabolism of sphingomyelin by sphingomyelinases (E.C.3.1.4.12) [57]. Sphingomyelinases exist in three forms: acidic, neutral, and alkaline, the latter also known as NPP7. These enzymes hydrolyze the phosphocholine headgroup of sphingomyelin to generate ceramide and phosphocholine. Ceramide can directly influence cell physiology or be phosphorylated by ceramide kinase and is metabolized to sphingosine-1-phosphate by the sequential action of ceramidase and sphingosine kinase [57].

SMPD1 encodes acid sphingomyelinase (ASM), expressed in Paneth [58] and endothelial cells of the small intestine. ASM is activated by Fas/Apo-1 [59], CD28 [60], and IL-1 signaling [61], as well as by ionizing radiation [16]. Ceramide generation by ASM in irradiated cells can induce apoptosis [16].

There are four different mammalian neutral sphingomyelinase (nSMase) enzymes, with nSMase2 being the predominant enzyme located in the Gogi and plasma membrane [62]. Similar to ASM, nSMases are activated by cytokines. Activation can occur following TNF-α binding to the TNF-α receptor-1, IL-1β binding to the IL-1 receptor, and interferon (IFN)-γ expression [62]. nSMase is also activated by irradiation, resulting in cell apoptosis [15].

Ceramide-induced apoptosis is initiated by several pathways [63]. Ceramide can inhibit phosphoinositide-3-kinase (PI3K) and Akt/PBK signaling pathways, which in turn can activate BAD, a proapoptotic effector. Additionally, ceramides activate protein phosphatase 2A (PP2A), which dephosphorylates and inactivates BCL2 [63]. Ceramides also form mitochondrial microdomains that promote BAX-mediated pore formation, resulting in mitochondrial outer membrane permeabilization (MOMP) [64, 65]. An additional mechanism involves the ability of ceramide to bind to Glu84 of the voltage-dependent anion channel 2, stabilizing mitochondrial BAX and BAK to form pores and induce MOMP [63].

A landmark study by Paris et al. [66], identified a correlation between radiation-mediated ceramide generation, lamina propria microvasculature apoptosis, and GI syndrome-mediated lethality in mice. Radiation doses sufficient to induce GI-ARS resulted in death within ≤10 days, which could not be prevented by bone marrow transplantation. Epithelial cell apoptosis was evaluated in cells located at crypt cell positions 1–8 [67], whereas endothelial cell apoptosis was quantified in the lamina propria. Extensive endothelial cell apoptosis occurred in the first 4 h after administering radiation doses sufficient for inducing the GI syndrome (≥15 Gy). In contrast, epithelial cell apoptosis occurred several hours after endothelial cell death [66]. This sequence suggested that GI-ARS is initiated by endothelial cell apoptosis.

Experiments using ASM-null and wild-type mice in a 129/Sv: C57BL genetic background [16, 66, 68] were used to determine whether there was a causal relationship between ceramide generation, endothelial apoptosis, and GI syndrome. Mice were administered whole-body irradiation (16 Gy). In ASM-null mice, microvascular apoptosis was significantly reduced, crypt-villus histology and length were preserved [69], and there was a significant increase in mouse survival [66]. Epithelial apoptosis was not inhibited in these mice. The experiment was repeated using C3H/FeJ ASM, Bax, or Bak wild-type or null mice and in 129/Sv/C57BL Bak wild-type or Bak null mice exposed to 15 Gy with autologous bone marrow transplantation (BMT) [70]. Loss of BAK or ASM in C3H/FeJ or 129/Sv/C57BL mice significantly reduced endothelial apoptosis. Microcolony assays revealed that crypt survival increased in BAK- and ASM-null mice (dose-modifying factor of 10%). The loss of BAK or BAX did not diminish crypt epithelial apoptosis. A radiation dose of 15 Gy administered to C3H/FeJ ASM wild-type mice reduced their lifespan to 5 days, while irradiated (15 Gy) C3H/FeJ ASM-null mice survived for >120 days.

The lifespan of C57BL Bax wild-type or null mice was approximately 5.6 days following a radiation dose of 15 Gy, even though the loss of Bax reduced endothelial apoptosis. In Bax wild-type C57BL mice with BMT, a radiation dose of 15 Gy reduced the mice’s lifespan to 6–9 days. Although this was a statistically significant increase in lifespan, one might conclude that the loss of Bax had a very small effect on the lifespan of irradiated C57BL mice [70]. This conclusion aligns with findings from experiments using C57BL Tie2Cre Bak1^-/-^;Bax^FL/-^ and C57BL Tie2Cre Bak1^-/-^;Bax^FL/+^ mice [71].

The p53 upregulated modulator of apoptosis (PUMA) is a critical mediator of both p53-dependent and independent apoptosis [72]. Qui et al. [72] investigated endothelial and intestinal epithelial apoptosis in PUMA wild-type and global knockout (null) mice following the administration of 15 or 18 Gy of irradiation. In wild-type mice, only 6% of the crypt cells (positions +3 to +9) underwent apoptosis 4–24 h after treatment with 18 Gy of irradiation (See Fig. 2B in Ref. [72]). Epithelial cell apoptosis was suppressed in irradiated PUMA-null mice. In contrast, approximately 15% of endothelial cells underwent apoptosis at 4 h post irradiation in both wild-type or PUMA-null mice (See Figures 3B and 3F located in Ref. [72]). The lifespan of PUMA-null mice was significantly enhanced compared with that of wild-type mice following irradiation. These results were interpreted to indicate that PUMA-mediated epithelial cell apoptosis, rather than endothelial apoptosis, plays a critical role in GI-ARS. However, 4 h after irradiation, there was a substantial, non-apoptotic (50%) loss of CD105-expressing endothelial cells in either the wild-type or PUMA-null intestinal submucosa (See Fig 3I in Ref. [72]). Thus, in this experimental model, there was considerable endothelial loss that might have contributed to GI-ARS.

Further support for the role of ceramide in GI syndrome was provided by Rotolo et al. [73]., who developed a monoclonal antibody against ceramide. The antibody blocked the radiation-induced formation of ceramide-rich scaffolds on endothelial plasma membranes, which are associated with the initiation of cell apoptosis. Intravenous administration of the antibody suppressed lamina propria endothelial apoptosis, increased CBC stem cell survival, and provided substantial protection from GI syndrome-related mortality. Thus, the antibody phenocopied the results obtained in ASM-null mice. The results obtained using ASM null mice and the ceramide antibody are interpreted as establishing a causal relationship between ceramide formation, endothelial apoptosis, and GI syndrome initiation.

The ability of fibroblast growth factor 2 (FGF2/bFGF) to function as a radioprotector has been used to strengthen the argument that endothelial apoptosis is an early and critical event in GI syndrome initiation. Houchen et al. [74], reported that irradiation of FVB/N mice increased FGF2 expression in the lamina propria of the small intestine but not in crypt cells. To gain insight into the consequences of FGF2 expression, recombinant FGF2 was injected into mice prior to irradiation. Administration of FGF2 decreased microvascular cell apoptosis, increased intestinal crypt cell survival, as measured using a microcolony assay [17, 74], and reduced fatal GI syndrome [66].

FGF2 is one of the 22 FGFs [75] whose signaling influences cell proliferation, survival, migration, and differentiation [76]. It maintains stem cells in a pluripotent, undifferentiated state [77]. FGF2 binds to FGFR2IIIb, FGFR2IIIc, FGFR3IIIb, FGFR3IIIc, and FGFR4 [77]. FGFR3IIIc has a high affinity for FGF2 [78] and is expressed in epithelial crypt cells of the adult mouse jejunum [78, 79], as well as in endothelial cells [80]. FGF2 binding to the FRFR3 receptor in epithelial cells induces BCL-2 and MCL-1, thereby blocking the induction of apoptosis [81]. FGF2 can also inhibit apoptosis of epithelial cells independent of FGFR3 via membrane translocation [82]. However, further studies are needed to determine whether FGF2/FGFR3 activation inhibits radiation-mediated apoptosis in endothelial cells, crypt stem cells, or both.

Jalili-Firoozinezhad et al. [83]. developed a human “gut-on-a-chip” model to investigate radiation-induced endothelial and epithelial cell death. A radiation dose of 8 Gy resulted in the expected loss of villus architecture and reduced height. Similar to the mouse model, endothelial apoptosis occurred within 24 h of irradiation, while epithelial apoptosis was observed 48 h post irradiation. They also quantified the release of lactate dehydrogenase (LDH), a marker of plasma membrane injury. Irradiation resulted in the release of LDH by approximately 10% of epithelial and endothelial cells, respectively, in the first 24 h post irradiation. Gut-on-a-chip models were constructed without endothelial cells and exhibited significantly less villus blunting post-irradiation compared with chip models containing both endothelial and epithelial cells. These findings suggest that endothelial cells are a primary target of radiation-induced intestinal injury.

The above studies compared endothelial cell apoptosis with epithelial cell apoptosis, supporting their conclusions concerning the role of endothelial cells in the development of GI-ARS. However, several seminal studies have revealed that GI-ARS is independent of p53-mediated apoptosis; p53-null mice experience significantly reduced survival after GI irradiation compared to wild-type mice even though epithelial apoptosis is suppressed. Knockout of the p21 gene also increases sensitivity to GI irradiation compared to wild-type mice. Conversely, over-expression of p53 diminishes sensitivity. The interpretation is that loss of p53/p21-mediated cell cycle checkpoints in proliferating epithelial cells results in rapid cell progression into mitosis. These cells do not have sufficient time to fully repair DNA damage and thus enter mitosis with unrepaired or miss-repaired DNA that triggers a mitotic catastrophe. Cell cycle checkpoint activation in p53/p21 in wild-type mice extends DNA repair capacity, reducing the number of cells entering mitosis with unrepaired DNA [71, 84, 85]. Thus, comparisons of apoptosis rates between these two cell compartments may be inappropriate. In summary, experiments that utilized several strains of irradiated mice other than C57BL mice support the hypothesis that endothelial cell death is an early event and that inhibiting ceramide generation genetically or inhibiting ceramide microdomain formation with antibodies extends lifespan. The human gut-on-a-chip model supports this hypothesis. The ceramide hypothesis underscores the importance of endothelial cell apoptosis as a contributor to GI-ARS development alongside epithelial crypt cell injury.

## Crypt recovery after irradiation

As early as 1990, Potten and Loeffler [86] proposed the existence of two populations of crypt cells: four to sixteen stem cells responsible for maintaining homeostasis and 30–40 transit-amplifying cells functioning as “potential/rescue/revival” stem cells after radiation injury. Analysis of crypt cell apoptosis in the small intestine following cytotoxic X-ray irradiation demonstrated that just one to three surviving cells were sufficient to repopulate a crypt. These surviving cells were postulated to be pluripotent stem cells [87, 88].

Several studies have used lineage tracing techniques and/or single-cell RNA sequencing to identify distinct reserve/rescue/revival cell populations. Genes, such as Bmi1, Hopx, Clu, or Msi1 and others located in the +4 area are examples of proteins interpreted to mark cells that can repopulate irradiated crypts [89–91]. The concept developed from these studies was that irradiation resulted in the loss of Lgr5+ cells; after irradiation-induced injury reserve/rescue/revive cells were induced to form Lgr5+ stem cells. These cells subsequently regenerated crypt cells via upward differentiation and migration. Beumer and Clevers [54] reviewed the extensive literature on +4 cell-mediated regeneration. They noted that quiescent reserve/rescue/revive gene markers are broadly expressed or that cells with these markers are biased toward secretory differentiation. The paradigm shifting experimental approaches used by Malagola et al. [55], have now clearly identified cells capable of regenerating crypts following irradiation as Lgr4+ isthmus progenitor cells. Whether cells at +4 should be included as isthmus progenitor cells needs further investigation.

Current research is now focusing on the molecular signals that inform cells to initiate crypt regeneration following irradiation and other stresses. For example, Fink et al. used transposase-accessible chromatin sequencing coupled with high-throughput sequencing (ATAC-seq) to investigate genome accessibility coupled with multi-omics. They discovered that the ability of crypt cells to regenerate resides in multiple cells located along the crypt axis, including differentiated cells. A key finding was that regeneration-dependent genes reside in a chromatin-accessible state, enabling chromatin remodeling that depends on TGF-β, Hippo, as well as p53 and TNFAIP8 innate immune signaling [92]. TP53 plays a crucial role in crypt regeneration. Regenerating cells exhibit “fetal-like reversion” and are enriched in p53 target genes. TP53 promotes a “fetal-like reversion” that is required for crypt regeneration [93]. TP53 expression is essential for suppressing IL12/MHC class II signaling, which drives T cell activation and inflammation [94]. In addition to uncovering the molecular signals that drive regeneration, the question of whether hierarchy exists among regenerative cell populations remains open.

When quantifying crypt cell apoptosis, Potten [88] identified a hypersensitive subpopulation consisting of less than 10 cells per crypt, characterized by an initial slope with a Do = 0.1 Gy. The Do is defined as the dose that results in an average of one lethal event occurring in a target volume. An event is defined as “an excitation, ionization, or ion cluster [95]. To understand the anatomical location of these hypersensitive cells, it is important to note that Potten et al. assumed that the cells at the crypt base consisted solely of Paneth cells (Supplementary Fig. S1A). However, a comparison of their findings with the current understanding of crypt anatomy shown in Supplementary Fig. S1B implies that these hypersensitive cells are located at positions +4 and above. Potten [88] also identified a more resistant population at a Do of 1 Gy. These studies revealed that CBC Lgr5+ cells were not sensitive to radiation. A similar conclusion was obtained by others [96], who employed crypt microcolony assays using Lgr5-LacZ transgenic mice.

Withers and Elkind [97] demonstrated that the mouse jejunum has a remarkable capacity for repair and regeneration. Microcolony and macrocolony survival assays were performed on irradiated jejunals. This assay quantifies crypt regeneration by surviving crypt cells and thus is directly related to GI-ARS life span studies. The log-linear dose-response curves for crypt regeneration showed an exponential slope across six decades of survival, with a Do of 1 Gy [98]. The mean lethal dose (1/Do) is the average lethal dose absorbed by a cell. This analysis indicates that the average lethal dose was the same for every cell that contributed to the exponential portion of the survival curve, and as reported [37, 55], it can be assumed that these are “isthmus progenitor” cells at positions +4 to +13 with regeneration potential. The DNA repair capacity of crypt cells, measured by the quasi-threshold dose (Dq) obtained from microcolony survival curves, is approximately 4.3 Gy [99], demonstrating that crypt cells indeed have a large repair capacity. Crypt regeneration is sex-dependent, with male mice exhibiting a greater degree of sensitivity than female mice [100]. Age also affects sensitivity, with aging mice being more sensitive than younger mice [101]. Future studies should explore how sex and age impact isthmus progenitor-mediated crypt regeneration following irradiation.

## Conclusions

ARS develops under specific conditions: the majority of the body must absorb a substantial radiation dose that penetrates internal organs and is delivered over a very short period. In humans, the GI syndrome develops at doses as low as 6 Gy. A dose of ≥10 Gy will result in death within 10 days. Patients with GI-ARS experience a prodromal phase characterized by nausea, vomiting, anorexia, and fatigue, followed by a latent stage and subsequently, a manifestation stage, during which symptoms reappear. Death or recovery occurred during the final stage.

The small intestine, folded into the plicae circularis, is divided into the duodenum, jejunum, and ileum. The villi and crypts within the small intestine house various cell types, including Paneth, crypt-based columnar, transit-amplifying, enterocytes, goblet, enteroendocrine, and tuft cells. Crypt-villus regeneration occurs every 3–5 days and is a consequence of a high rate of crypt cell proliferation, balanced by the sloughing-off of the fully differentiated villi. Fgfbp1 and Lgr4 have been identified as genes associated with high stem cell potency and are expressed in cells located in “isthmus progenitor” cells (positions +4 to +13). These progenitor cells expand bidirectionally, functioning as both homeostatic and rescue stem cells. Radiation dose-response curves indicate that radiation sensitivity is independent of location within the crypt.

Research supports the hypothesis that GI-ARS is initiated by two critical events: ceramide-induced endothelial injury and mitotic catastrophe-induced crypt cell death. This leads to subsequent inflammatory responses, including activation of caspase-1, and increased expression of IL1β, TNFα, and NLRP3. Transepithelial resistance decreases with the loss of the epithelial tight junction barrier. Developing a comprehensive understanding of vascular regeneration and identifying the biochemical signals required to initiate and drive isthmus progenitor cell-mediated crypt regeneration are essential for the development of effective medical countermeasures against GI-ARS.

## Supplementary information

Legend for Supplemental Figure S1

Supplemental Figure S1
