# Supplementary material for: Gastrointestinal acute radiation syndrome: current knowledge and perspectives
Source: Cell Death Discov. 2025 May 14;11:235. doi: 10.1038/s41420-025-02525-6 (PMC12078527; doi:10.1038/s41420-025-02525-6)
Supplement: Supplementary file 1 — Legend for Supplemental Figure S1 [file 41420_2025_2525_MOESM1_ESM.docx]

**Supplemental Figure S1:**

S1A: Illustration of the crypt-villus structure adapted from Ijiri and Potten. British Journal of Cancer 1983, 47:175.

S1B: Illustration of the crypt-villus structure adapted from Gehart and Clevers , Nature Reviews | Gastroenterology & Hepatology 2019, 16:19.

Developed using BioRender
