# Supplementary figures and images for: Gastrointestinal acute radiation syndrome: current knowledge and perspectives
Source: Cell Death Discov. 2025 May 14;11:235. doi: 10.1038/s41420-025-02525-6 (PMC12078527; doi:10.1038/s41420-025-02525-6)

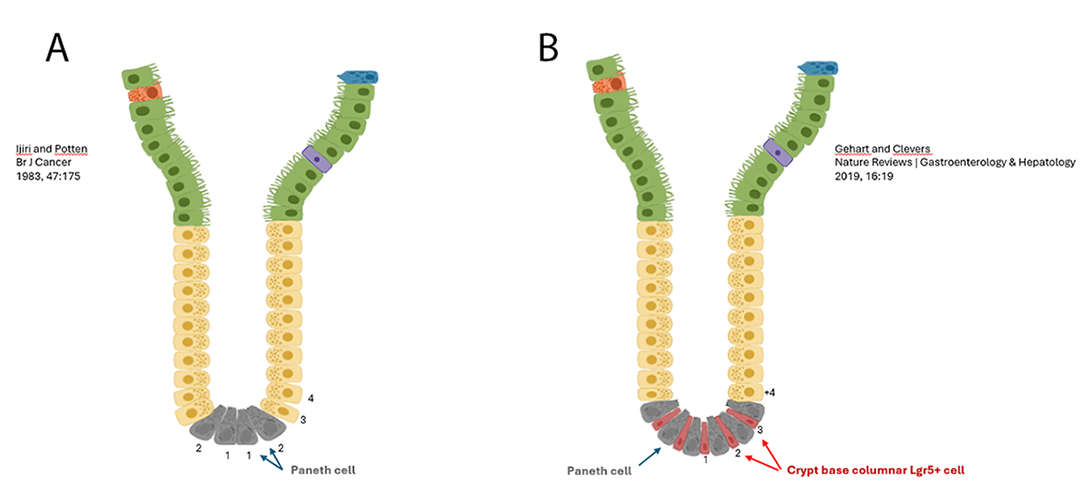

Supplement: Supplementary file 2 — Supplemental Figure S1 [file 41420_2025_2525_MOESM2_ESM.tif]
